# Epidemic Anthrax in the Eighteenth Century, the Americas

**DOI:** 10.3201/eid0810.020173

**Published:** 2002-10

**Authors:** David M. Morens

**Affiliations:** * National Institutes of Health, Bethesda, Maryland, USA

**Keywords:** anthrax, epidemiology, medical history

## Abstract

Anthrax has been described as a veterinary disease of minor importance to clinical medicine, causing occasional occupational infections in single cases or clusters. Its potential for rapid and widespread epidemic transmission under natural circumstances has not been widely appreciated. A little-known 1770 epidemic that killed 15,000 people in Saint-Domingue (modern Haiti) was probably intestinal anthrax. The epidemic spread rapidly throughout the colony in association with consumption of uncooked beef. Large-scale, highly fatal epidemics of anthrax may occur under unusual but natural circumstances. Historical information may not only provide important clues about epidemic development but may also raise awareness about bioterrorism potential.

In late 2001, anthrax bioterrorist attacks in the United States prompted considerable commentary on how little is generally appreciated about the transmissibility of *Bacillus anthracis*. Textbooks have long described anthrax as a veterinary disease of minor medical importance, attributing most human infections to occupational exposures, now less common in industrialized nations. Because anthrax is usually recognized in single cases or small clusters, its potential for rapid and broad dissemination to humans under natural circumstances has not been widely appreciated. Such a potential would have implications for both epidemics and bioterrorism.

An obscure report claims that an explosive 1770 epidemic of what was called *charbon* killed 15,000 persons in Saint-Domingue (modern Haiti). The brief description of this epidemic, written by historian Michel-Placide Justin (1), is unknown to most physicians and historians. The epidemic began shortly after an earthquake near Port-au-Prince on June 3, 1770, devastating the city and much of the western end of the island. With bakeries, stores, storehouses, and many or most of the buildings and homes in major towns destroyed, and with the consequent escape of slaves who typically obtained, transported, and prepared food for themselves and the colonists, famine was a serious threat. Trade regulations in force at the time specifically restricted importation of meats or salted fish. Justin describes the situation as follows:

"…The unfortunate slaves in the north of Saint-Domingue therefore experienced the most frightful famine. The dependencies of Fort Dauphin, that of Gros-Morne, [and] of Jean Rabel, were devastated. Codfish being entirely unavailable, the Spaniards, whose *hattes* [presumably a form of the Spanish “hato”, meaning "cattle ranch"] or pastures were being thinned out daily by a terrible epizootic ["épizootie"], sought to salt or smoke all their ill or dead animals; and they [then] brought them into French establishments. These meats, known as *tassau* in the colonies, which the Negroes avoided eating when they could get [uncontaminated] salted beef and codfish, spread to the slaves the communicable agent ["germe"] of the disease with which they [the meats] were infected ["infectées"]. A type of epidemic disease ["peste"], called anthrax ["charbon"], spread throughout all the neighboring dwellings of the Spaniards or the routes they frequently used, and in those where the Negroes had bought this *tassau*. Within six weeks, more than 15,000 white and black colonists perished of this terrible disease, and its ravages did not stop until the government, the magistrates, and the inhabitants themselves had joined all of their efforts to repel the scourge introduced into the colony by Spanish greed.

 “But the numerous and rapid deaths caused by the disease were not all: at least 15,000 Negroes perished of hunger, and the escape of slaves increased in the northern dependency, causing serious fear for the security of the colony…" (1).

Although sketchy, this report of possible epidemic anthrax contains interesting details. It notes the precipitating circumstances of an ongoing epizootic and a sudden change in diet to uncooked—smoked or salted—beef. The report also discloses that outbreak investigation linked the distribution of contaminated beef to the geographic spread of human disease. These associations appear consistent with intestinal anthrax, a disease associated with high mortality. However, exact means and determinants of gastrointestinal transmission were not described. Salted or smoked meat likely would have been eaten without cooking, as was then the custom. Since anthrax spores are resistant to 140°F and to a wide range of chemical treatments, the failure of salting or smoking to destroy them would not be surprising.

Apparently the overall mortality in the epidemic was high, although the figure of 15,000 deaths may have been only an estimate. Epidemic and vital events data from the epidemic were probably obtained by French officials and sent to Paris, but I am not aware that such data, if they still exist, have been identified by historians. Neither attack rates nor case-fatality rates have been documented from this or similar anthrax epidemics in the same era, although eighteenth-century observers were nearly unanimous in indicating a high or universal death rate from intestinal anthrax.

Had the epidemic occurred several years later, it might have received more thorough official attention. In 1776, Félix Vicq-d'Azyr set up a system of epidemic surveillance and outbreak investigation that operated in France and her colonies until 1794 (2). Two of its "correspondent" proto-epidemiologists, Drs. Arthaud and Girard, were in place in Saint-Domingue to report on epidemics by November 1777 and February 1778, respectively. Six years later, Saint-Domingue's *Cercle des Philadelphes*, of which Benjamin Franklin became a member, had been established and had also begun to study medical and veterinary diseases. Publications of this and other societies describe Haitian epidemics in the early 1770s, but none mention the 1770 outbreak.

The identity of the disease described in Justin's report can be questioned. I have translated the report’s designation of *charbon* as "anthrax," the term corresponding to modern anthrax. But in 1770, 10 years before epizootic anthrax had been reasonably well described by Chabert (3) and 95 years before its microbial cause was fully demonstrated by Davaine (4), the term *charbon* (“charcoal”) was sometimes applied nonspecifically to other human diseases producing skin lesions, including not only dark or violaceous lesions of any sort but also plague and smallpox. Justin's sources for the 1770 epidemic report are unknown, but he did not begin to write it until 1822 or 1823, by which time human and animal anthrax had become better understood.

Other possibilities for the cause of the epidemic seem less likely. Smallpox epidemics periodically swept Caribbean islands (e.g., Barbados in 1751, causing a serious case of smallpox in future U.S. president George Washington [5]). However, it was well known that smallpox did not cause epizootics in cattle, and French officials would not likely have mistaken such a familiar disease. Aside from its clear clinical picture, any epidemic that spared past smallpox victims would have been immediately noted by Europeans, all of whom knew their own status with regard to smallpox susceptibility. Moreover, by 1770 many colonists and slaves had been variolated, making recognition of epidemic smallpox even more likely.

In 1801, American proto-epidemiologist (and future lexicographer) Noah Webster speculated that the 1770 epidemic "must have been the real plague" (6), what we now call “bubonic plague.” This speculation seems to have been based on his discovery during 1799–1801 of 30-year-old gazette accounts, which he did not, unfortunately, cite. Webster might also have been influenced by description of the disease as a type of "peste," a word which, in the 1770s, could mean either an epidemic disease of any kind or the specific disease now known as plague, caused by *Yersinia pestis*. However, neither bubonic nor pneumonic plague is consistent with a cattle epizootic or an association with beef distribution. A "fatal angina" or "distemper" (also described as a "sore throat") appears to have been epidemic in the Caribbean in 1770 (7,8), but in that era such terms usually indicated either diphtheria or streptococcal pharyngitis (9), neither of which causes fatal epizootics. Rabies was introduced into Saint-Domingue about 1776 (10) but seems entirely inconsistent on clinical and epidemiologic grounds.

In rare post-Webster medical references to the 1770 epidemic, anthrax has not been questioned. For example, a passing reference in a medical text by anthrax authority Carl von Heusinger (11), published in 1850, agrees on anthrax, a diagnosis subsequently accepted without comment in George Fleming’s 1871–1882 history of epizootics (12) and in James Law's 1885 review of "malignant pustule" (13).

Also notable with regard to the epidemic's identity are 1775 reports claiming that a less severe epizootic of the same disease recurred in Saint-Domingue in 1772, spread to Guadeloupe, then recurred again in Saint-Domingue in 1773, 1774, and 1775. These subsequent epidemics affected cattle and caused, in humans, both cutaneous lesions, associated with inoculation, and gastrointestinal diseases, associated with ingestion (10,14). These reports and others published by members of the *Cercle des Philadelphes* appear to be excellent early descriptions of anthrax. The author of one of them (14), proto-epidemiologist Charles Arthaud, sent information from the 1774–75 epizootic to colleagues at the recently opened veterinary school at Alfort. Anthrax had also been occurring episodically in Europe. However, given the school's receipt of such detailed epidemic reports from the Caribbean colonies, including the clearest documentation to date of the means of cattle-to-human transmission, the Saint-Domingue epizootic/epidemic and related ones must have played a role in the classical first characterization of anthrax by Alfort’s director Philibert Chabert in 1780 (3). Chabert's treatise seems to draw directly on the Saint-Domingue reports forwarded by his colleagues, one of whom was Chabert's mentor and the founder of the French veterinary schools, Henri Bertin (10).

Historians have occasionally speculated that large-scale anthrax epidemics occurred in the past (e.g., one of the Pharaonic plagues described in the biblical book of Exodus, occurring around 1491 BC, and an epidemic in seventeenth century Europe [15]), but evidence is weak. Anthrax has also been proposed as the cause of the notorious "Plague of Athens" in 430 BC, a proposition consistent with signs and symptoms of intestinal anthrax in humans and possibly epizootic involvement of dogs and birds of prey (16).

Like the Athenian epidemic—considered by Friedrich Prinzing to be anthrax and included by him among the classic "diseases resulting from wars" (17)—the Saint-Domingue epidemic occurred during a time of upheaval, coming as it did during a devastating earthquake, impending famine, slave revolt, trade wars, and ongoing discord between French and Spanish colonists. The possibility of biological warfare in either epidemic, however, seems remote. Several years before the 1770 epidemic, during the French-Indian War, British general Lord Jeffrey Amherst wrote a letter in which he discussed giving smallpox-contaminated blankets to North American Indians, and some historians believe the British actually did so (18). By 1770, the French-Indian Wars were over; little would have been gained on any side by harming both French and Spanish colonists, as well as slaves and free residents.

Historical evidence from "natural experiments," such as the 1770 Saint-Domingue epidemic, should be considered in public health efforts to prevent disease re-emergence and increase awareness about bioterrorism potential. In developing countries, single cases and small clusters of severe and fatal intestinal anthrax still occur, often in association with butchering ill animals to obtain consumable and salable meat before the animals die. Such occurrences underscore the importance of efforts to maintain a safe food supply. If historically recorded and widespread intestinal anthrax transmission via broadly distributed meats is accepted as accurate, this 200-year-old evidence would reinforce the need to be vigilant in maintaining safeguards to prevent accidental and purposeful contaminations of food products.
